# Hyaluronidase treatment of synovial fluid is required for accurate detection of inflammatory cells and soluble mediators

**DOI:** 10.1186/s13075-021-02696-4

**Published:** 2022-01-08

**Authors:** Hilde Brouwers, Johannes Hendrick von Hegedus, Enrike van der Linden, Rachid Mahdad, Margreet Kloppenburg, René Toes, Martin Giera, Andreea Ioan-Facsinay

**Affiliations:** 1 Department of Rheumatology, Leiden University Medical Center (LUMC), Leiden, The Netherlands; 2 Department of Orthopedics, Leiden University Medical Center, Leiden, The Netherlands; 3 Department of Orthopedics, Alrijne Healthcare Group, Leiden, The Netherlands; 4 Center for Proteomics and Metabolomics, Leiden University Medical Center (LUMC), Leiden, The Netherlands

**Keywords:** Synovial fluid, Hyaluronidase, Rheumatoid arthritis

## Abstract

**Background:**

Synovial fluid (SF) is commonly used for diagnostic and research purposes, as it is believed to reflect the local inflammatory environment. Owing to its complex composition and especially the presence of hyaluronic acid, SF is usually viscous and non-homogeneous. In this study, we investigated the importance of homogenization of the total SF sample before subsequent analysis.

**Methods:**

SF was obtained from the knee of 29 arthritis patients (26 rheumatoid arthritis, 2 osteoarthritis, and 1 juvenile idiopathic arthritis patient) as part of standard clinical care. Synovial fluid was either treated with hyaluronidase as a whole or after aliquoting to determine whether the concentration of soluble mediators is evenly distributed in the viscous synovial fluid. Cytokine and IgG levels were measured by ELISA or Luminex and a total of seven fatty acid and oxylipin levels were determined using LC-MS/MS in all aliquots. For cell analysis, synovial fluid was first centrifuged and the pellet was separated from the fluid. The fluid was subsequently treated with hyaluronidase and centrifuged to isolate remaining cells. Cell numbers and phenotype were determined using flow cytometry.

**Results:**

In all patients, there was less variation in IgG, 17-HDHA, leukotriene B_4_ (LTB_4_), and prostaglandin E_2_ (PGE_2_) levels when homogenization was performed before aliquoting the SF sample. There was no difference in variation for cytokines, 15-HETE, and fatty acids arachidonic acid (AA), eicosapentaenoic acid (EPA), and docosahexaenoic acid (DHA). Between 0.8 and 70% of immune cells (median 5%) remained in suspension and were missing in subsequent analyses when the cells were isolated from untreated SF. This percentage was higher for T and B cells: 7–85% (median 22%) and 7–88% (median 23 %), respectively.

**Conclusions:**

Homogenization of the entire SF sample leads to less variability in IgG and oxylipin levels and prevents erroneous conclusions based on incomplete isolation of synovial fluid cells.

**Supplementary Information:**

The online version contains supplementary material available at 10.1186/s13075-021-02696-4.

## Background

Synovial inflammation is a symptom of many rheumatic musculoskeletal diseases such as rheumatoid arthritis (RA), juvenile idiopathic arthritis (JIA), spondyloarthritis (SpA), osteoarthritis (OA), and systemic lupus erythematosus (lupus). Synovial joints contain a synovial lining (synovium) consisting primarily of synovial fibroblasts and a synovial cavity containing SF.

During inflammation, immune cells such as macrophages, lymphocytes, and neutrophils infiltrate the joint and SF accumulates. The cells and soluble mediators present at these local sites of inflammation are of interest to unravel disease pathophysiology and are therefore often studied.

Synovial fluid cytokines have been of interest for many years and more recently, lipid-derived inflammatory mediators were studied in RA and OA [1–6]. Next to the interest in soluble mediators, frequencies, and phenotype of specific T and B cell subsets, monocytes and NK cells were determined in a wide range of rheumatic musculoskeletal diseases [7–24]. In addition to experimental research, synovial fluid is also used for diagnostic purposes to obtain information about antigen-specific antibody levels or white blood cell (WBC) count [25–27].

Synovial fluid is often aspirated from the inflamed joints of arthritis patients to reduce discomfort and to make room to inject therapeutics or to perform diagnostics, as part of standard clinical care. Therefore, it is easier accessible for research than synovium, which can only be obtained via a more invasive procedure such as arthroplasty or synovial biopsy. Synovial fluid provides lubrication to the joint and acts as a transport medium for nutrients and cells. It consists of an ultrafiltrate of plasma containing many different proteins including high amounts of hyaluronan. Hyaluronan is produced by the synovial fibroblasts and is present in a 10^5^ higher concentration in synovial fluid compared to plasma [28]. Hyaluronan forms dense mesh networks in the synovial fluid, which make the fluid viscous. Due to this viscosity, handling synovial fluid in a laboratory setting is challenging. To overcome this problem, synovial fluid can be treated with hyaluronidase, which breaks down the dense mesh network of hyaluronan fibers. Indeed, a few reports, analyzing equine SF or SF from non-inflammatory conditions, have shown that technical issues can occur when analyzing untreated SF [29–31]. For instance, it was shown that treating SF with hyaluronidase before cytokine analysis improves cytokine recovery in a polystyrene, but not magnetic bead, Luminex assay [31]. In addition, hyaluronidase treatment is routinely performed before proteomic and metabolomic analysis as this is required to prevent clogging of the mass spectrometers [5, 32]. However, to date there are no reports on the importance of hyaluronidase treatment as part of the standard processing protocol for SF and this treatment is therefore not routinely used in arthritis research. For example, hyaluronidase treatment is not mentioned in more than half of the abovementioned studies investigating SF immune cells in inflammatory joint diseases [1, 3, 6–10, 12-15, 20, 33]. In daily practice, SF is collected, aliquoted, and stored before analysis to be able to analyze soluble mediators in multiple patients at once. Subsequently one aliquot per patient is then used to measure soluble mediators. However it is unclear whether the levels of soluble mediators are comparable between the aliquots when the aliquots were taken and stored before hyaluronidase treatment. Moreover the effect of hyaluronidase treatment on the recovery of cells from SF is unclear and there is no consensus whether inflammatory cells should be analyzed before or after hyaluronidase treatment.

In this study, we investigated whether homogenization of SF sample is required for the reproducibility of IgG, cytokine, and lipid measurements. In addition, we performed flow cytometric analysis on cells isolated from treated and untreated SF.

## Methods

### Patients

Synovial fluid was obtained via knee aspiration from 27 arthritis patients (26 rheumatoid arthritis and 1 juvenile idiopathic arthritis patient) visiting the rheumatology outpatient clinic of the LUMC as part of standard clinical care. Written informed consent was obtained from all these donors. Anonymized leftover synovial fluid of two osteoarthritis patients was collected using a syringe before knee-replacement surgery performed at the departments of orthopedic surgery at the LUMC and the Alrijne hospital in Leiden. The study was approved by the local ethical committee.

### Processing of synovial fluid for molecular measurements

On the same day of knee aspiration, the fluid was divided in two sets of 3–5 aliquots ranging between 300 μL and 1 mL to be able to fit the fluid in a heat block with vortex modus. 1 mg/mL hyaluronidase from bovine testis (Sigma-Aldrich) was added in a 1:11 ratio. Hyaluronidase was suspended just before use in phosphate-buffered saline (PBS, B.Braun). SF was vortexed for 5 min and subsequently incubated for 25 min at 37°C. Next, the first set of aliquots (set 1) was directly centrifuged for 10 min at 931x*g*, while the second set (set 2) was first pooled together before centrifugation. After centrifugation, the pooled set was divided again in 35 individual aliquots. Of each of the aliquots in set 1 and set 2, 100 μL supernatant was transferred to a glass vial (Agilent Technologies) together with 294 μL methanol (Fluka LC-MS CHROMASOLV grade, Sigma-Aldrich) and 6 μL internal standards (50 ng/mL leukotriene B_4_-d4, prostaglandin E_2_-d4, 15-HETE-d8, and 500 ng/mL of docosahexaenoic acid-d5, all from Cayman Chemicals). Argon gas was added and the samples were stored at −80°C. Similarly, 200 μL supernatant was also stored at −20°C for immunoglobulin and IL-8 analysis. For IL-6, IL-10, CXCL1, CXCL5, and TNFα analysis, SF was centrifuged for 10 min at 931x*g* on the day of knee aspiration and supernatant was stored directly at −80°C. At the day of analysis, SF was thawed to room temperature and treated as described above for the fresh SF.

### ELISA

Interleukin 8 (IL-8) and IgG ELISA were performed according to manufacturer’s protocol (Invitrogen, BD Biosciences, and Bethyl Laboratories, respectively).

### Luminex

Bio-Plex pro reagent kit III and Bio-Plex Pro Human IL-6 set (171BK29MR2), IL-10 set (171BK32MR2), TNF-α (171BK55MR2), CXCL1 (171BK22MR2), and CXCL5 (171BK14MR2) were purchased from Bio-rad. The cytokines and chemokines were determined according to the manufacturer’s protocol. SF samples were measured on the Bio-Plex 200 system (Bio-Rad), and analysis was done using Bio-plex Manager 6.2 Software (Bio-Rad).

### Lipidomic analysis of synovial fluid

LC-MS/MS-based lipid mediator and oxylipin profiling were carried out as described elsewhere [4]. A QTrap 6500 mass spectrometer (Sciex) was used, coupled to a Shimadzu Nexera LC30-system including auto-sampler and column oven (Shimadzu). The column was a Kinetex C18 50 × 2.1 mm, 1.7 μm, protected with a C8 pre-column (Phenomenex). LC-MS/MS peaks were integrated with manual supervision, and the areas were corrected to corresponding IS using MultiQuant™ 2.1 (Sciex). For quantitation, the multiple reaction monitoring (MRM) transitions and collision energies (CE) were used together with calibration lines for quantification. Calibration lines were constructed using 15-HETE, 17R-HDHA, leukotriene B_4_, prostaglandin E_2_, arachidonic acid, docosahexaenoic acid, and eicosapentaenoic (Cayman Chemicals)

### Flow cytometry analyses of synovial fluid cells

For general analysis, the fluid was centrifuged for 10 min at 931x*g* and cells were collected and analyzed. The supernatant was treated with hyaluronidase as stated above and subsequently centrifuged for 10 min at 931x*g* to isolate remaining cells. For the effect of hyaluronidase treatment on cell marker expression, the SF was diluted 20x in PBS and divided in two. One sample was treated with hyaluronidase (treated), and the other sample was treated similarly, but without the addition of hyaluronidase (untreated). Isolated cells were resuspended in PBS and filtered through a 70-μm cell strainer before flow cytometry. For general cell characterization, cells were stained with anti-CD3 (AF700, clone UCHT1), anti-CD14 (FITC, clone M5E2), anti-CD15 (APC, clone HI98), anti-CD16 (PE, clone B73.1), anti-CD19 (PerCp/Cy5.5, clone SJ25C1), and anti-CD45(APC/Cy7, clone 2D1). The gating strategy is depicted in supplementary figure 1. For additional characterization into CD4^+^ T cells and CD8^+^ T cells, cells were stained with anti-CD3 (PE, clone SK7), anti-CD4 (APC, clone SK3), and anti-CD8 (FITC, clone SK1). For the effect of hyaluronidase treatment on cell marker expression, cells were stained with anti-CD3 (Pacific Blue, clone SK7), anti-CD4 (APC, clone SK3), anti-CD8 (FITC, clone SK1), anti-CD19 (APC/Cy7, clone SJ25C1), anti-CD44 (PE/Cy7, clone G44-26), and anti-CD69 (PE/CF594, clone FN50). The positivity for CD44 and CD69 was determined using isotype controls IgG2bk (PE/Cy7, clone 27-35) and IgG1 (PE/CF594, clone X40). For additional characterization into naïve B cells, memory B cells and plasmablasts/cells, cells were stained with anti-CD3 (Pacific Blue, clone SK7), anti-CD14 (Pacific Blue, clone M5E2), anti-CD19 (APC/Cy7, clone SJ25C1), anti-CD20 (AF 700, clone 2H7), and anti-CD27 (PE/Cy7, clone M-T271). For gating strategy for the T cell and B cell subsets, see supplementary figure 2.

All antibodies were from BD except for the anti-CD20 which was from Sony Biotechnology (USA, CA, San Jose). For all analysis, dead cells were excluded using DAPI (Molecular Probes) and cells were quantified using Flow count Fluorospheres (Beckman Coulter). Cells were measured on a LSR Fortessa (BD) and were analyzed with FACSDiva Software (BD).

### Statistical analyses and calculations

Wilcoxon signed rank tests were performed to evaluate significance between groups in all figures. Coefficient of variation (CV) was calculated by dividing the standard deviation of a group of measurements by the mean and expressing this as a percentage.

## Results

### Effect of hyaluronidase on soluble mediator measurements

**Fig. 1:**
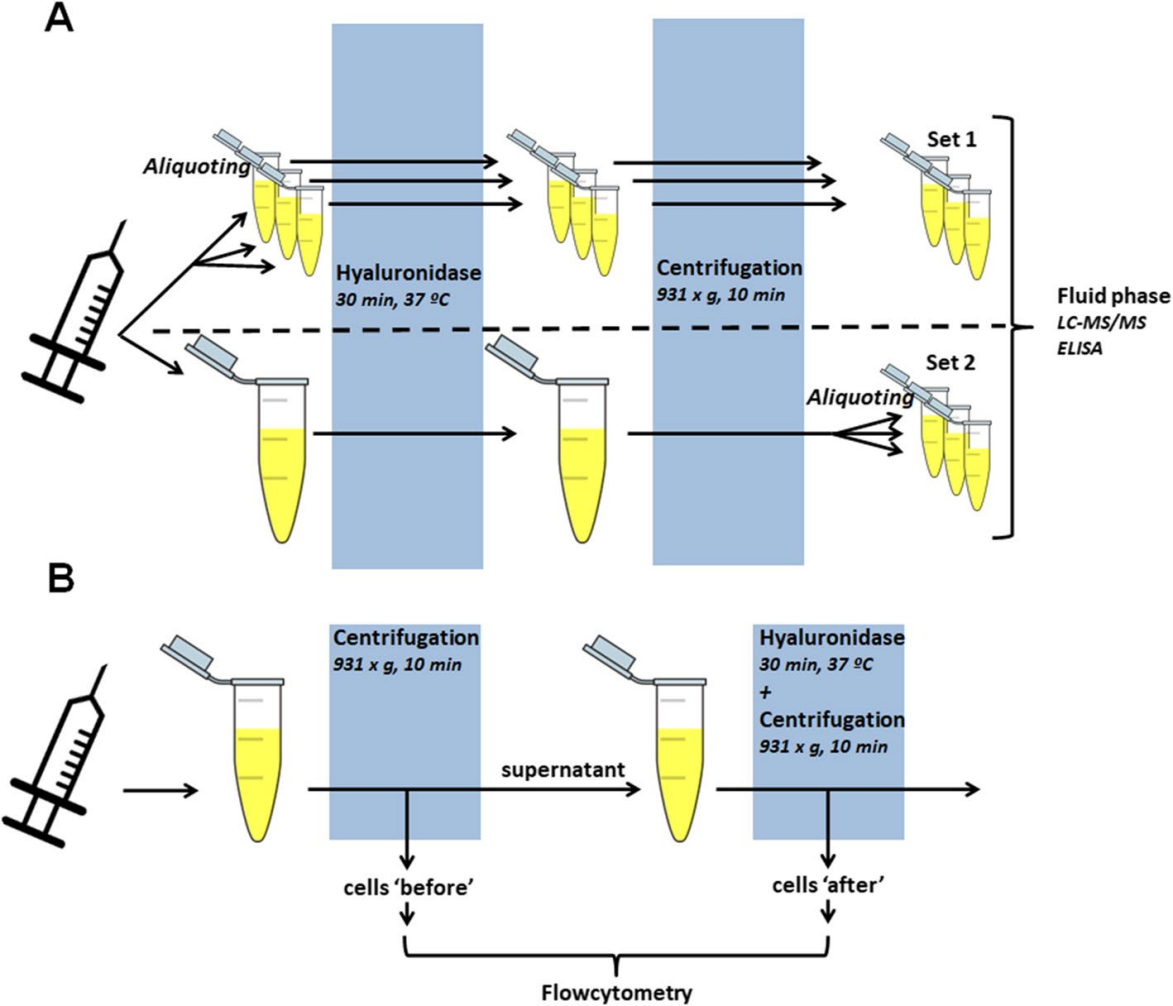
Schematic overview of the experimental setup. **A** Half of the synovial fluid was divided into aliquots while the other half was kept as a whole. The SF was treated with hyaluronidase after which it was centrifuged. Set 1 was kept as separate aliquots during the whole procedure and set 2 was divided in aliquots directly after centrifugation. **B** Synovial fluid was centrifuged and the pelleted cells are the “before” cells. The supernatant was treated with hyaluronidase and subsequently centrifuged. The pelleted cells are the “after” cells

**Fig. 2:**
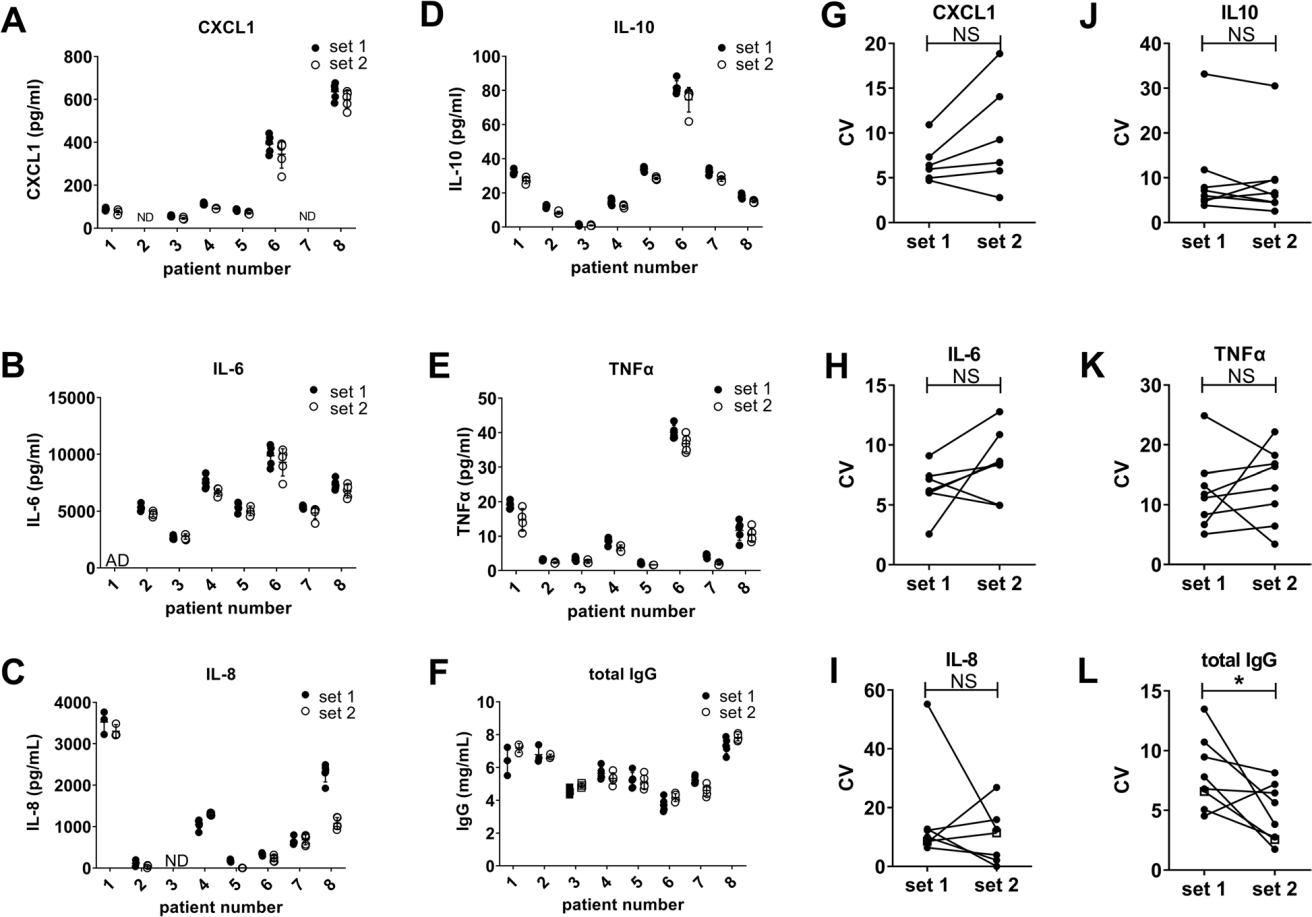
Hyaluronidase treatment lowers variability in IgG levels. **A**–**F** CXCL1, IL-6, IL-8, IL-10, TNFα, and total IgG levels measured by ELISA or Luminex. Dots are RA patients, and the OA patient is represented by squares. Each point represents one aliquot. Means with SD are shown. IL-8 and total IgG were analyzed in a separate set of 8 patients compared to CXCL1, IL-6, IL-10, and TNFα. ND not detected. AD above detection limit. **G**–**L** Coefficients of variation (CV) are depicted for each set of aliquots. Each line represents one patient. *n*=6–8 patients. Dots are RA patients, and the OA patient is represented by open squares Wilcoxon signed rank test was performed. *P<0.05

**Fig. 3:**
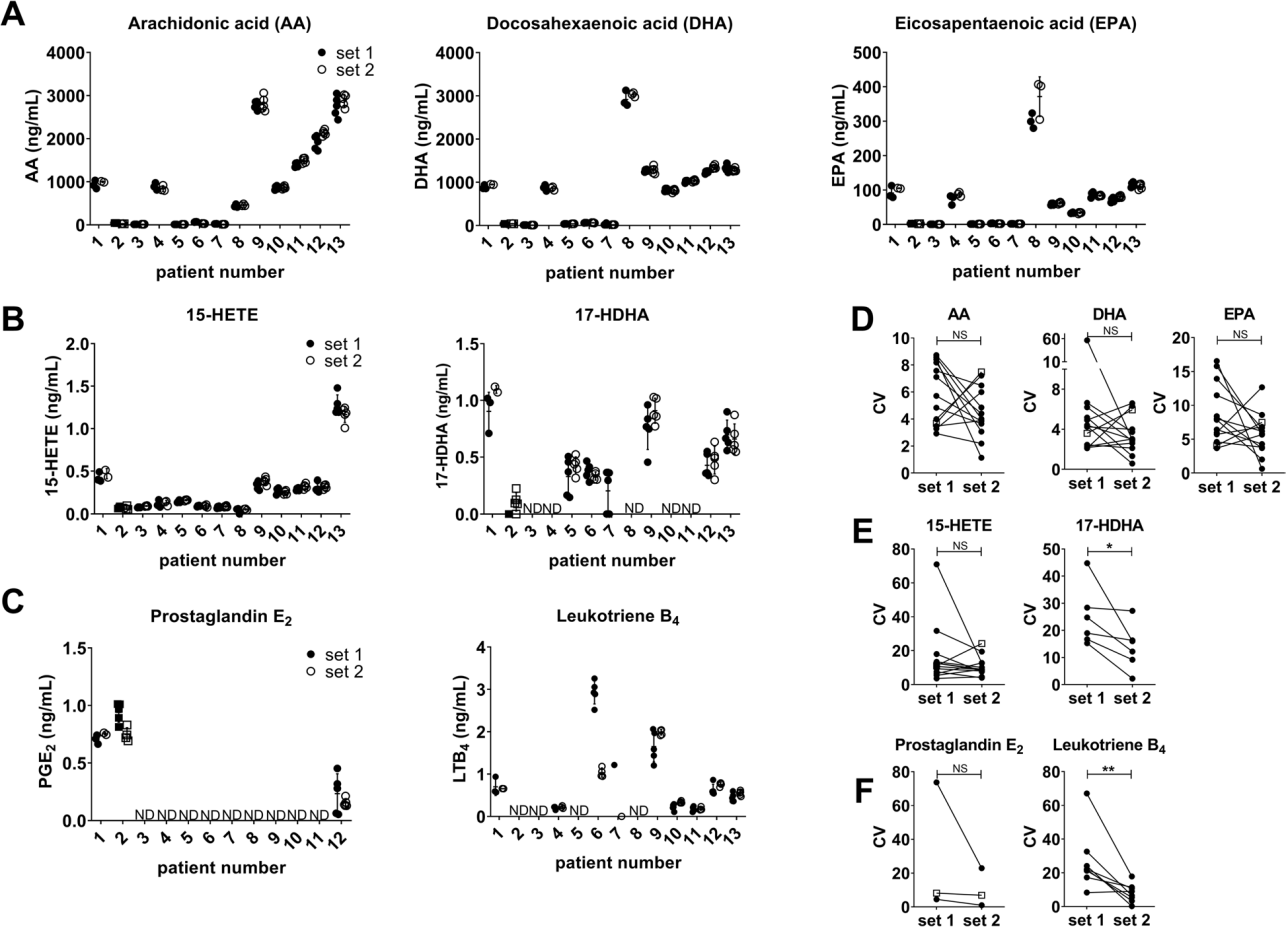
Reproducibility of lipid measurements improves by aliquoting after hyaluronidase treatment. The concentrations for each set of aliquots are depicted for **A** fatty acids and **B** monohydroxylated products and **C** oxylipins. The CVs for each set of aliquots are depicted for **D** fatty acids and **E** monohydroxylated products and **F** oxylipins. *n*=13 patients and each line represent one patient. Dots are RA patients, and the OA patient is represented by squares. No line or ND means not detected. Wilcoxon signed rank test was performed. *P<0.05; **P<0.01

To test the whether the concentration of inflammatory soluble mediators is evenly distributed in viscous synovial fluid, we treated each synovial fluid as depicted in Fig. 1A. We also treated set 1 with hyaluronidase to exclude the possibility that the viscosity hampers soluble mediator detection as was shown before [31]. We measured CXCL1, CXCL5 IL-6, IL-8, IL-10, TNFα, and total IgG levels in the aliquots of set 1 and set 2 in eight arthritis patients. Levels of the cytokines differed considerably between patients (Fig. 2A–E). The data of CXCL5 is not shown as this mediator was only detected in two out of eight patients. To assess intra-assay variation, the CV was calculated for each set of aliquots of the patients with detectable soluble mediator levels. There was no difference in CV between the two sets of samples for CXCL1, IL-6, IL-8, IL-10, TNFα (Fig. 2B). IgG levels were stable between the different patients and in contrast to the cytokines, hyaluronidase treatment before aliquoting resulted in lower CVs for IgG measurements (Fig. 2F, L). In addition to measurements of large protein structures as cytokines and antibodies, we investigated small molecules, such as fatty acids and oxidized lipids (Fig. 3A–C). Hyaluronidase treatment before aliquoting did not improve CV values for AA, DHA, and EPA (Fig. 3D). These fatty acids can be converted to monohydroxylated fatty acids such as 15-HETE and 17-HDHA, derived from AA and DHA, respectively. Hydroxylated products can in turn be converted to highly bioactive oxylipids like PGE_2_ and LTB_4_. In contrast to the CV values of fatty acids, the values for 17-HDHA and LTB_4_ improved significantly when fluids were treated with hyaluronidase before aliquoting (Fig. 3E, F).

### Cellular measurements benefit from hyaluronidase treatment

**Fig. 4:**
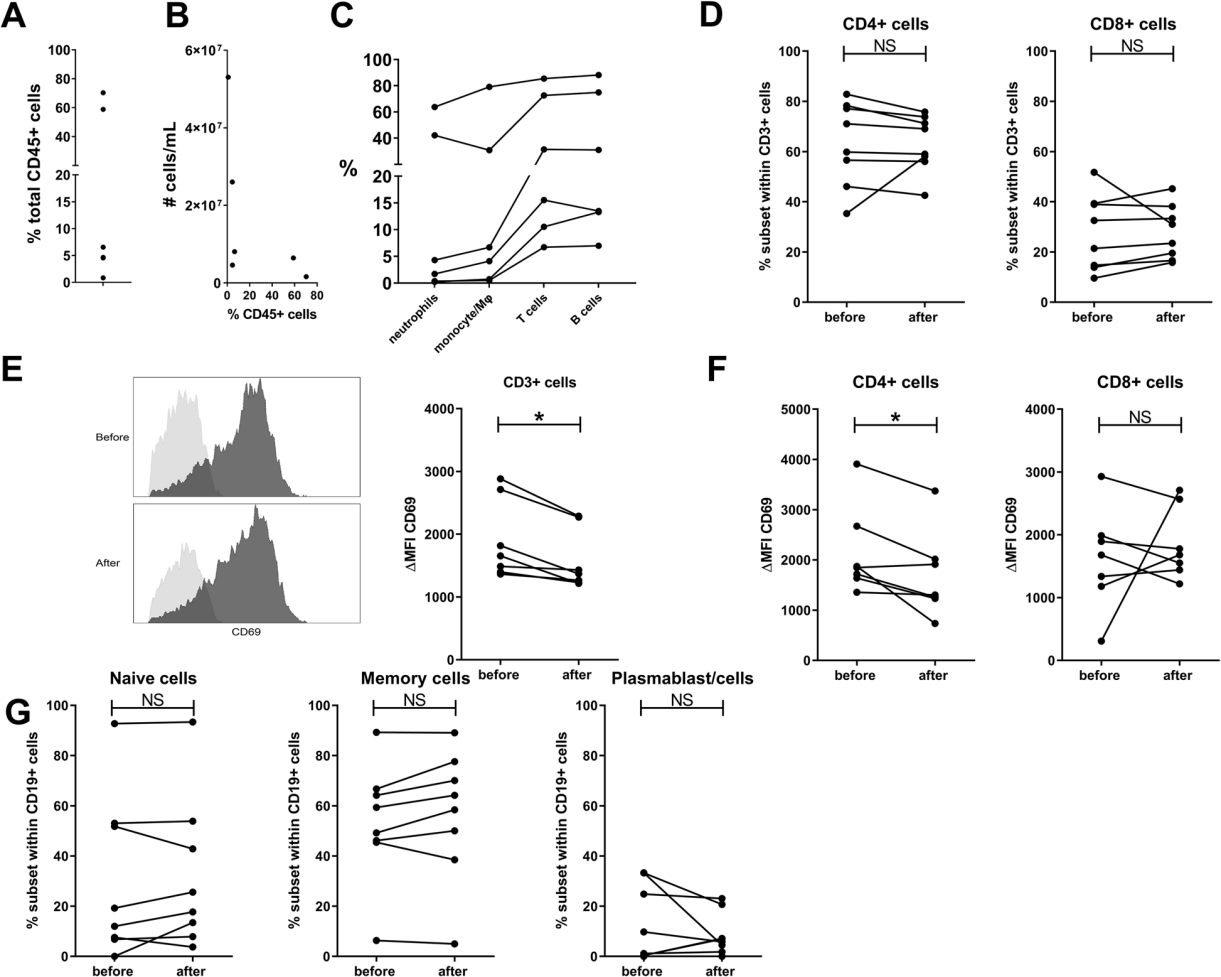
Hyaluronidase treatment is essential for an unbiased analysis of the cellular composition of synovial fluid. **A**, **C** Percentage of cells obtained after hyaluronidase treatment relative to total (before hyaluronidase + after hyaluronidase) number of cells. Cell subsets are defined as follows: neutrophils are CD15^+^CD16^+^, monocyte/macrophages are CD15-CD14^+^, T cells are CD3^+^, and B cells are CD19^+^. Each dot or line represents one patient. *n*=5–6 patients. **B** Percentages from A plotted against the number of live cells per mL in the total SF (before hyaluronidase + after hyaluronidase) SF determined using flow count beads. **D** Percentages of CD4^+^ and CD8^+^ cells present within the CD3^+^ cell population obtained before hyaluronidase treatment (before) or after (after). **E** CD69 expression on CD3^+^ cells. Flowcytometry plot of one representative donor is shown together with the summary of all patients. The expression of the cellular marker is indicated by ΔMFI which is defined as MFI of the antibody staining (dark gray)—MFI of the isotype control (light gray). **F** CD69 expression on CD4^+^ and CD8^+^ cells. **G** Percentage of naïve (CD20^+^CD27−) and memory (CD20^+^CD27^+^) B cells, as well as plasmablast/plasma cells (CD20-CD27^+^) present within the CD19^+^ population before and after hyaluronidase are depicted. Each line is one patient. *n*=7–8 patients. Wilcoxon signed rank test was performed.

We hypothesize that the viscosity of SF impairs the recovery of immune cells during isolation. To test this, readily-accessible cells (before fraction) were first isolated from SF by centrifugation. Thereafter, the supernatant, i.e., hyaluronan-rich fraction (after fraction), was treated with hyaluronidase and centrifuged again, to isolate remaining cells (Fig. 1B). Depending on the patient, up to 70% of the CD45^+^ cells could be isolated from the supernatant after hyaluronidase treatment (Fig. 4A). This percentage did not correlate with the total number of cells present in the fluid (Fig. 4B). Interestingly, mostly T and B cells were recovered from the hyaluronan-rich (after) fraction (Fig. 4C). Further characterization of T cell subsets revealed no difference in CD4^+^ or CD8^+^ T cell percentages between the before and after fractions (Fig. 4D). Surprisingly, however, the cells in the after fraction displayed a less activated phenotype, reflected by a lower expression of CD69^+^ (Fig. 4E). This effect was primarily observed in the CD4^+^ T cells (Fig. 4F). There was no difference in the percentage of CD69^+^ cells in CD3^+^, CD4^+^, or CD8^+^ T cells between the before and after samples (data not shown). The hyaluronidase treatment itself does not affect CD69 expression (Supplementary figure 3B). We could not detect any differences in either naïve B cell, memory B cell, or plasmablast/plasmacell percentages between the fractions (Fig. 4G).

### Smaller immune cells are retained in the hyaluronan-rich supernatant

**Fig. 5:**
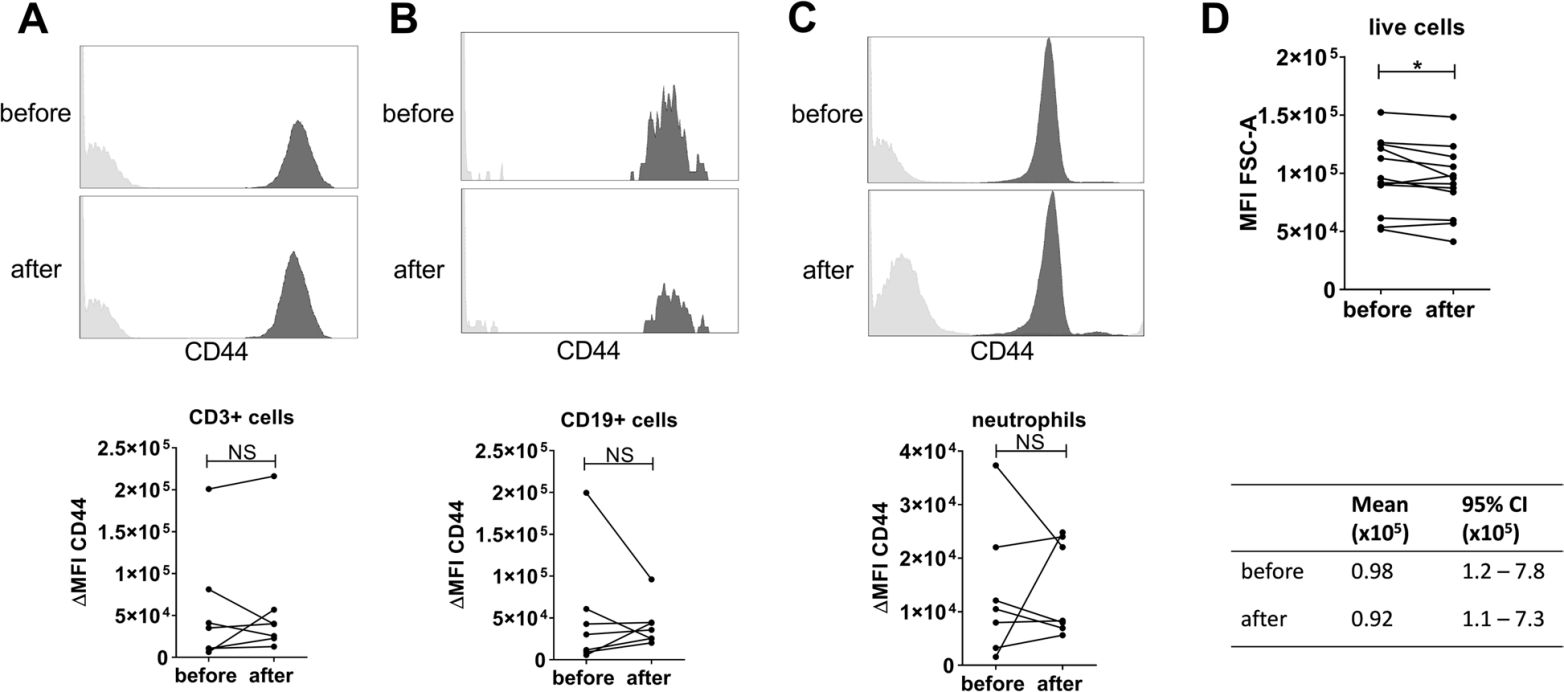
Smaller immune cells remain in SF after centrifugation without hyaluronidase. **A–C** Treatment of SF and the analysis of cells were performed as described in Fig. 4, except for neutrophils, which were identified based on FSC/SSC. CD44 expression on CD4^+^ and CD8^+^ T cells, CD19^+^ B cells, and neutrophils. Flowcytometry plot of one representative donor is shown together with the summary of all patients. Each line represents a patient. *n*=7 patients. Wilcoxon signed-rank test was performed. **D** MFI values FCS-A of live cells determined by flowcytometry as described in Fig. 4. Each dot represents one patient. *n*= 12 patients. Mean and 95% shown in lower panel. Wilcoxon signed-rank test was performed

To gain more insight into the possible mechanism underlying the retention of certain lymphocytes in the hyaluronan mesh, we investigated expression of the hyaluronan receptor CD44 and the size of the immune cells in the SF supernatant [34]. We hypothesized that cells with a higher expression of CD44 could be preferentially retained in the untreated SF. However, there was no difference in CD44 expression on lymphocytes and neutrophils (Fig. 5A–C). In control experiments, the hyaluronidase treatment itself did not affect CD44 expression (Supplementary figure 3A). Interestingly, smaller cells are preferentially retained in the untreated SF (Fig. 5D).

## Discussion

Hyaluronidase treatment of SF is not routinely used in rheumatology research; however, our results show the critical importance of homogenization of the synovial fluid sample to obtain reproducible and accurate immunological measurements. We show that IgG titers, 17-HDHA levels, and pro-inflammatory LTB_4_ levels become significantly less variable when SF is treated with hyaluronidase before aliquoting. In addition, hyaluronidase treatment of SF prior to cell isolation highly increased isolated cell numbers and therefore positively affects the accuracy of subsequent cell analysis.

Several joint diseases are characterized by the presence of auto-antibodies such as RF and ACPA in RA and ANA and anti-dsDNA antibodies in SLE [25, 26]. However, the mere presence of the autoantibodies in circulation does not directly indicate a role for these antibodies in joint inflammation or destruction. Therefore, researchers are investigating presence, relative abundance, and specificity of these antibodies in the synovial fluid of, e.g., arthritis patients [33–36]. In these experiments, it is important to accurately determine the presence and specificity of autoantibodies in the joint. Our data shows that total IgG levels are not evenly distributed throughout a synovial fluid sample as measurements were less variable when taking aliquots from homogenized synovial fluid compared to when they were taken from untreated synovial fluid. We did not analyze presence or abundance of antibodies of certain specificity, but as they are present in much lower concentrations than total IgG, higher variability between aliquots might more easily result in erroneous significant differences between patient samples.

In addition to total IgG levels, 17-HDHA and pro-inflammatory LTB_4_ levels were also less variable when SF was aliquoted after homogenization. Although we could not show a significant reduction of CVs of PGE_2_ concentrations, as PGE_2_ was only detected in 3 out of 13 patients, the 3 positive patients show a similar trend as for LTB_4_. Fatty acid concentrations were stable between the two sets of aliquots, this could be due to a high abundance compared to oxylipins (100–3000 ng/mL versus 0.1–1.5 ng/mL) or better dispersion in SF as we hypothesize that the oxylipins might interact more with their environment [5].

It was shown before that reproducibility of IL-6 and IL-8 measurements in SF by multiplex (Luminex) analysis are affected by hyaluronidase treatment [31]. In contrast, we show that the reproducibility of IL-6, IL-8 and also CXCL1, IL-10, and TNFα measurements is equal between aliquots taken before or after hyaluronidase treatment of SF. However, this difference could easily be explained by the differences in experimental set-up. We have treated both sets of aliquots with hyaluronidase (Fig. 1A) wherease Jayadev et al. compared untreated versus treated SF. In that study, accurate measurements of IL-6 and IL-8 might easily be hampered by the viscousity of the SF explaining their finding. They indeed show that the viscosity of the untreated aliquots hampers cytokine measurements in the Luminex assay.

Since immune cells in local tissues are of great interest in understanding disease pathology, we have compared cell populations isolated from untreated and treated SF. Up to a surprisingly high percentage (70%) of cells are often excluded from analyses when cells are isolated from untreated SF. Since specifically T and B cells are excluded, resulting data are likely highly biased. Moreover, we show that within the CD4^+^ T cell populations, additional bias is introduced within the CD69^+^ cells. Based on our results, we hypothesize that specifically T and B cells are excluded based on their cellular size, since we could not confirm any increased interaction with the SF environment compared to other cell types. However, this hypothesis could be further tested through analysis of additional cell adhesion molecules. In addition, we could perform experiments using different sized beads to support our hypothesis of the involvement of cellular size.

## Conclusions

In summary, we show that homogenization of SF using hyaluronidase leads to less variability in oxylipin and IgG level measurements. In addition, homogenization of SF improves the recovery of immune cells during cell isolation. Based on our data, we advise researchers to perform hyaluronidase treatment on SF samples before aliquoting and subsequent analyses. Implementing hyaluronidase treatment in protocols using SF will likely result in improved reproducibility of soluble mediator measurements, new insights in the cell type ratios in joint diseases and even in the discovery of previously unidentified cell types in SF.

## Supplementary Information

**Additional file 1: Supplementary figure 1**. Gating strategy of experiments shown in Fig. 4A-C. **A)** Cells were gated separately from flow count beads on FSC-A/SSC-A and doublet cells were excluded by setting gates in FCS-W/FCS-H and SSC-W/SSC-H plots. Dapi staining was used to exclude dead cells and CD45+ cells were gated based on antibody staining. **B)** Neutrophil gates, monocyte gates and lymphocyte gates were set based on morphology on the FCS-A/SSC-A. **C)** The monocyte gate was plotted and contaminating CD15+ neutrophils were excluded after which CD14+ monocyte/macrophages were gated. **D)** The neutrophil gate is plotted and the neutrophils are characterized by CD15+ and CD16+ positivity The lymphocytes were plotted in panel **E)** and **F)** to be able to gate CD3+ T cells and CD19+ B cells respectively based on antibody staining.

**Additional file 2: Supplementary figure 2**. Gating strategy of experiments shown in Fig. 4D and E. **A)** Flow count beads, neutrophil gates and lymphocyte gates were set based on morphology on the FCS-A/SSC-A. Doublet cells were excluded by setting gates in FCS-W/FCS-H and SSC-W/SSC-H plots. Dapi staining was used to exclude dead cells and the live cell gate was plotted to gate CD3+ cells based on antibody staining. The CD3+ cells were further analyzed for CD4 and CD8 expression. B) Gates for Flow count beads, lymphocyte gate and single cells were set the same was a in panel A. Dead cells, CD3+ cells and CD14+ cells were excluded by gating the negative cells. These Dapi/CD3/CD14 negative cells were plotted and analyzed for CD19. CD19+ B cells were further analyzed for CD27 and CD20 to evaluate naïve B cell, memory B cell and plasmablast/plasmacell numbers.

**Additional file 3: Supplementary figure 3.** Hyaluronidase treatment does not effect cell marker expression. Synovial fluid was diluted 20x in PBS and divided in two. One sample was treated with hyaluronidase (treated) and the other sample was treated similar but without the addition of hyaluronidase (untreated). Cells were isolated by centrifugation and CD44 (**A**) and CD69 (**B**) expression was analyzed on various cell types in two donors (dashed versus closed line). **C**) Lineage marker expression is shown in two donors (stars versus dots). ΔMFI is calculated using the isotype control. Wilcoxon signed rank test was performed. n=2 donors.

## Data Availability

The datasets used and/or analyzed during the current study are available from the corresponding author on reasonable request.

## Abbreviations

AA: Arachidonic acid
CV: Coefficient of variation
DHA: Docosahexaenoic acid
EPA: Eicosapentaenoic acid
IL: Interleukin
JIA: Juvenile idiopathic arthritis
LTB_4_: Leukotriene B_4_
OA: Osteoarthritis
PGE_2_: Prostaglandin E_2_
RA: Rheumatoid arthritis
SF: Synovial fluid
SpA: Spondyloarthritis
WBC: White blood cell
