# Supplementary figures and images for: Hyaluronidase treatment of synovial fluid is required for accurate detection of inflammatory cells and soluble mediators
Source: Arthritis Res Ther. 2022 Jan 8;24:18. doi: 10.1186/s13075-021-02696-4 (PMC8742425; doi:10.1186/s13075-021-02696-4)

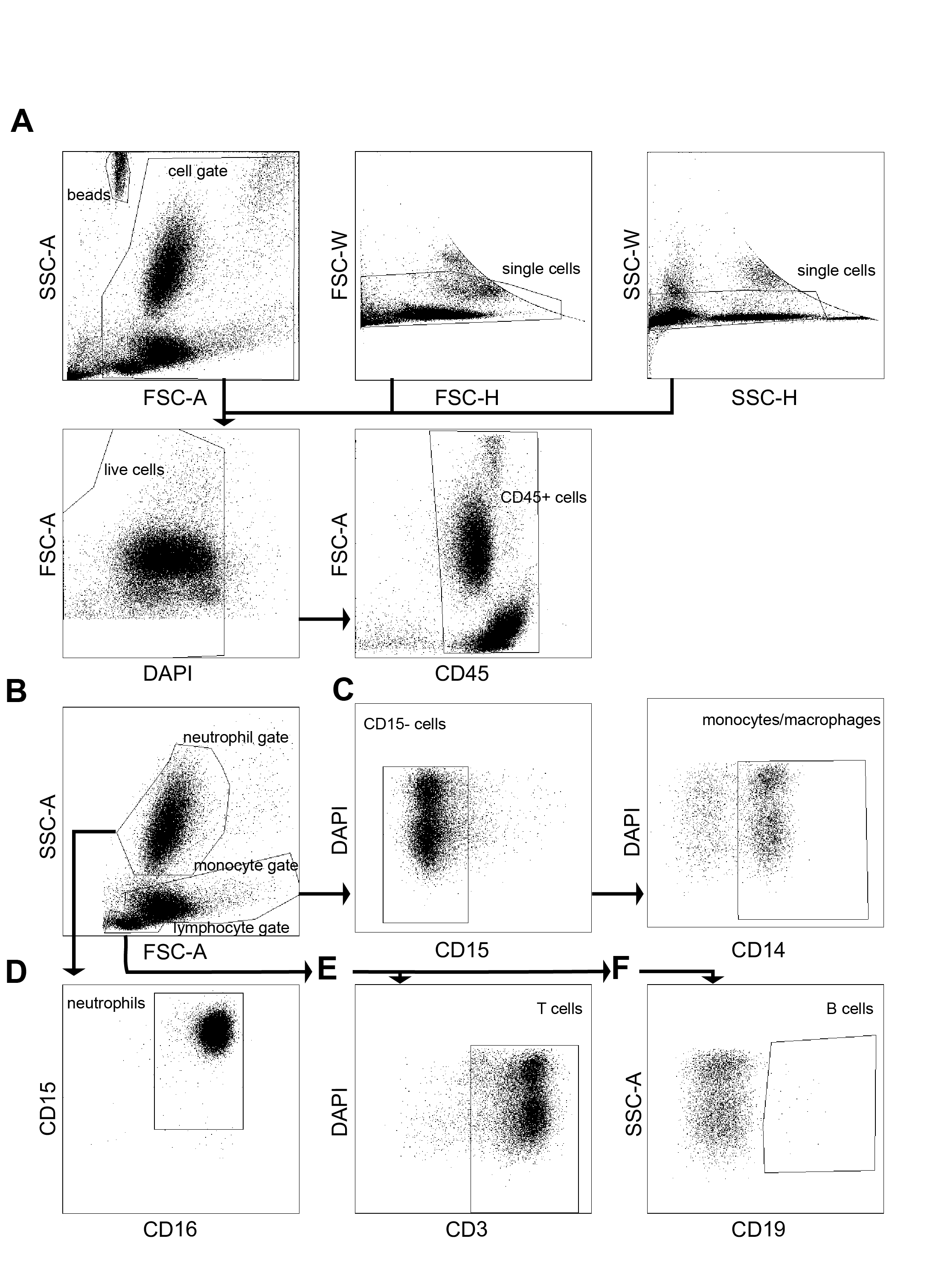

Supplement: Supplementary file 1 — Additional file 1: Supplementary figure 1. Gating strategy of experiments shown in Fig. 4A-C. A) Cells were gated separately from flow count beads on FSC-A/SSC-A and doublet cells were excluded by setting gates in FCS-W/FCS-H and SSC-W/SSC-H plots. Dapi staining was used to exclude dead cells and CD45+ cells were gated based on antibody staining. B) Neutrophil gates, monocyte gates and lymphocyte gates were set based on morphology on the FCS-A/SSC-A. C) The monocyte gate was plotted and contaminating CD15+ neutrophils were excluded after which CD14+ monocyte/macrophages were gated. D) The neutrophil gate is plotted and the neutrophils are characterized by CD15+ and CD16+ positivity The lymphocytes were plotted in panel E) and F) to be able to gate CD3+ T cells and CD19+ B cells respectively based on antibody staining. [file 13075_2021_2696_MOESM1_ESM.tif]

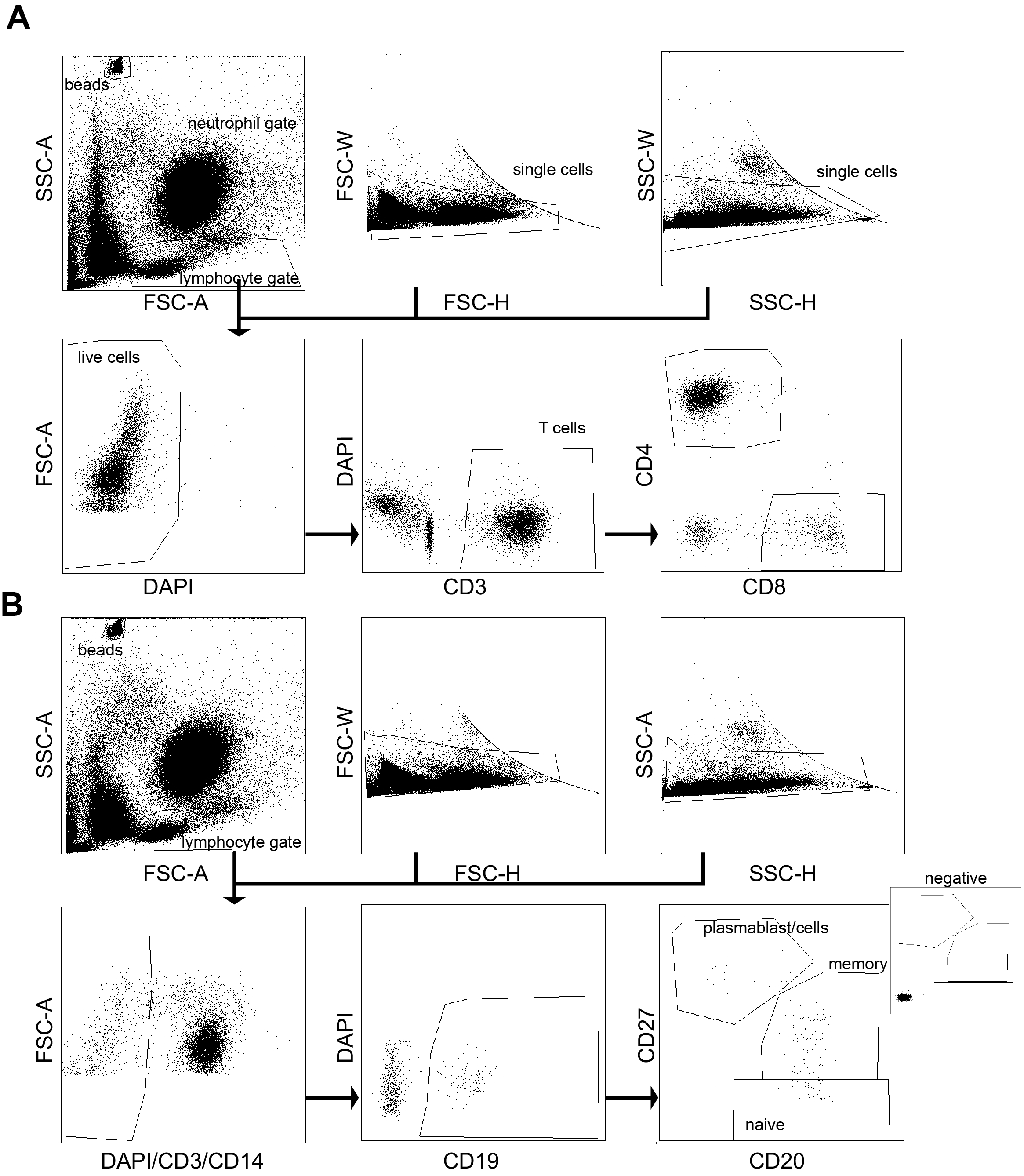

Supplement: Supplementary file 2 — Additional file 2: Supplementary figure 2. Gating strategy of experiments shown in Fig. 4D and E. A) Flow count beads, neutrophil gates and lymphocyte gates were set based on morphology on the FCS-A/SSC-A. Doublet cells were excluded by setting gates in FCS-W/FCS-H and SSC-W/SSC-H plots. Dapi staining was used to exclude dead cells and the live cell gate was plotted to gate CD3+ cells based on antibody staining. The CD3+ cells were further analyzed for CD4 and CD8 expression. B) Gates for Flow count beads, lymphocyte gate and single cells were set the same was a in panel A. Dead cells, CD3+ cells and CD14+ cells were excluded by gating the negative cells. These Dapi/CD3/CD14 negative cells were plotted and analyzed for CD19. CD19+ B cells were further analyzed for CD27 and CD20 to evaluate naïve B cell, memory B cell and plasmablast/plasmacell numbers. [file 13075_2021_2696_MOESM2_ESM.tif]

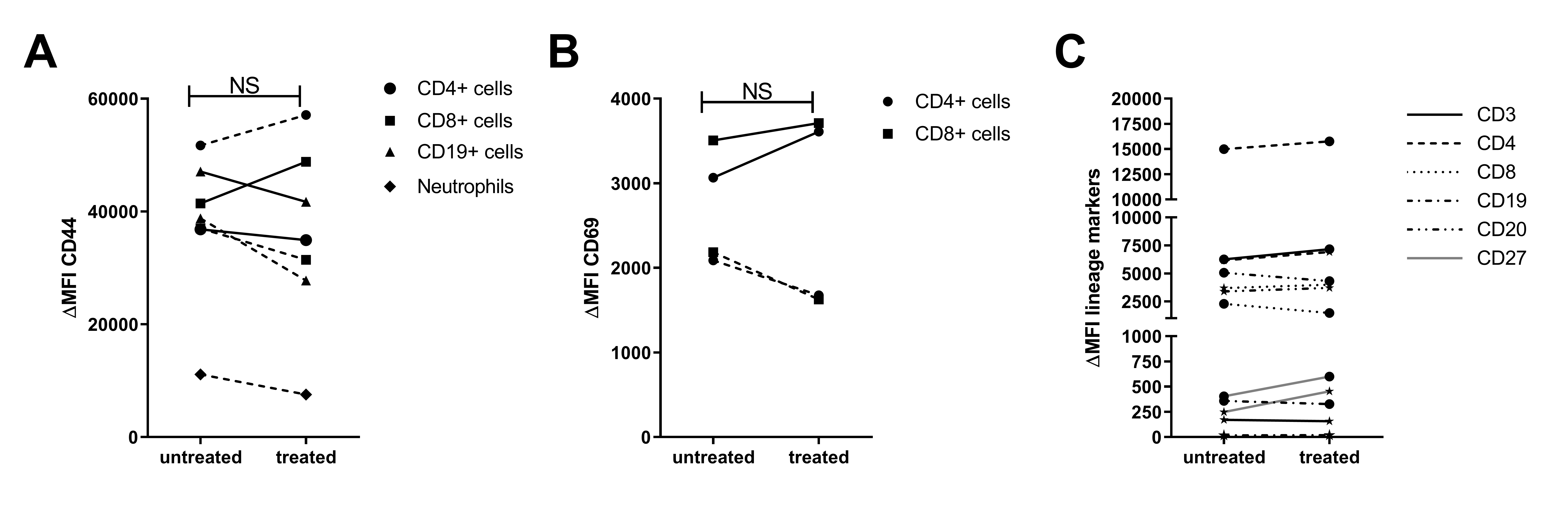

Supplement: Supplementary file 3 — Additional file 3: Supplementary figure 3. Hyaluronidase treatment does not effect cell marker expression. Synovial fluid was diluted 20x in PBS and divided in two. One sample was treated with hyaluronidase (treated) and the other sample was treated similar but without the addition of hyaluronidase (untreated). Cells were isolated by centrifugation and CD44 (A) and CD69 (B) expression was analyzed on various cell types in two donors (dashed versus closed line). C) Lineage marker expression is shown in two donors (stars versus dots). ΔMFI is calculated using the isotype control. Wilcoxon signed rank test was performed. n=2 donors. [file 13075_2021_2696_MOESM3_ESM.tif]
